# Performance-driven switched reluctance motor drive using multiport cascaded converter and advanced direct torque control scheme

**DOI:** 10.1038/s41598-026-45141-9

**Published:** 2026-04-13

**Authors:** M. Deepak, C. Santhakumar, K. Sathiyasekar, Bharatiraja Chokkalingam, Sanjeevikumar Padmanaban

**Affiliations:** 1 Department of Electrical and Electronics Engineering, KIT-Kalaignar Karunanidhi Institute of Technology, Coimbatore, India; 2 Department of Electrical and Electronics Engineering, K. S. R. College of Engineering, Tiruchengode, India; 3 Centre for Electric Mobility, Department of Electrical and Electronics Engineering, SRM Institute of Science and Technology, Kattankulathur, Chengalpattu, Tamil Nadu 603203 India; 4 Department of Electrical Engineering, Information Technology and Cybernetics, University of South-Eastern Norway, Kongsberg, Norway

**Keywords:** Switched reluctance motor, Modular cascade converter, Direct torque control, Torque ripple, Harmonic distortion, Energy science and technology, Engineering

## Abstract

The switched reluctance motor (SRM) drive exhibits inherent characteristics such as robustness, high efficiency, and fault tolerance that make it particularly well-suited for traction applications. However, SRM drives controlled by specialized converters often produce high torque ripples and harmonic distortion. On the other hand, conventional two-level converters for SRMs increase stress during switching operations. To address these challenges, this paper proposes an enhanced modular multiport cascade converter (MMCC) for SRM drives, aimed at minimising torque ripples and harmonic distortion. By increasing the number of voltage levels, the performance of the SRM drive is improved when combined with an enhanced direct torque control (DTC) strategy. The precise SRM model and the MMCC switching controlled by optimized DTC switching tables are integrated and evaluated using MATLAB/Simulink. The developed model analyzes input current and voltage harmonics across switches during drive operation. The proposed model behaviour for steady-state and dynamic performance is validated through speed, torque, and phase current measurements. A hardware demonstration of the SRM drive under various operating conditions further confirmed enhanced performance. The proposed converter achieves a 41.5% reduction in torque ripple compared to its conventional counterpart, thereby significantly enhancing dynamic performance and demonstrating strong suitability for variable drive applications.

## Introduction

Switched reluctance motors (SRMs) are poised to play a crucial role in the future of electric vehicles (EVs) traction systems due to their distinct advantages over traditional motor technologies. Unlike permanent magnet motors, SRMs do not rely on rare earth materials, which are expensive and have supply chain vulnerabilities. This makes SRMs more cost-effective and sustainable in the long run. Additionally, SRMs are known for their robust and simple construction, which translates to higher reliability and lower manufacturing costs^1^. Their ability to operate efficiently across a wide range of speeds and torques makes them highly adaptable to the varying demands of EV driving conditions^2^. Moreover, advancements in power electronics and control algorithms have significantly mitigated the issues of torque ripple and noise, which were earlier drawbacks of SRMs. As the automotive industry continues to seek more efficient, reliable, and sustainable solutions, SRMs are increasingly recognized as a promising candidate for the next generation of electric vehicle traction motors^3,4^. The main role of converters in SRM drives is to control the current flowing through the motor phases, thereby managing the motor’s torque and speed.

In the literature review^5,6^, typically, power electronic circuits switch the phase windings on and off in a precise sequence to create the desired magnetic field that drives the rotor, and converters play a crucial role in controlling the motor’s performance. This process involves converting the DC supply voltage to the appropriate phase currents needed for the SRM operation, creating more torque ripples. In SRM drives, high torque ripples and harmonics are due to the high voltage across the switches during their turn-on and turn-off periods. The conventional modular cascade converter phase sequence is operated with simple control techniques such as modulation control, hysteresis control, and PI control, providing high torque ripple for the SRM drive. In Refs. 7 and 8, high-frequency switching of converters in SRM drives can generate significant torque content. Additionally, harmonics can cause electromagnetic interference and affect the performance of the motor. In Refs. 9 and 10, high voltage across switches during operation can lead to increased voltage stress and increasing torque ripples. This can cause premature failures of noise, and vibrations in EVs disturb all four quadrants of operations. In Ref. 11, a conventional asymmetric converter with direct torque control (DTC) creates more torque ripples due to improper selection of voltage vectors and sector division. In addition, the DTC techniques adopted a different strategy to estimate the flux and torque used to control the switching operation of converters, which produce high torque ripple due to their band limits and switching frequency limits. This ripple results in vibrations and acoustic noise, which can reduce the comfort and perceived quality of electric vehicles. In Refs. 12 and 13, DTC with cascade converters creates harmonics that affect the power quality of the system, leading to inefficient power usage and potential overheating of components due to distorted current waveforms. In Ref. 14, DTC with torque and flux band limits the use of a two-phase excitation technique to reduce torque ripple and improve performance by mitigating harmonics. In Refs. 15, 16, the improper selection of DTC switching tables affects the steady state speed analyses of the sources of conducted high torque ripples in SRM drives. In Refs. 17, 18, model predictive DTC techniques with converter design that reduces torque ripples and minimizes harmonic content, enhancing the overall efficiency and reliability of SRM drives. However, the estimation of torque and flux, and the prediction of the control objective function, are more complicated to design and implement in real-time applications. In Ref. 19, SRM conventional converters and controllers integrated with electric vehicles to charge and discharge are used by multilevel cascade converters with high torque ripples due to improper switching of the phase sequence conduction and overlap periods.

The key contribution

- The mathematical model of non-linear SRM was developed with accurate parameters, flux linkage, current, rotor position, and torque.
- Enhancing the design of modular multiport cascade converters and switching techniques can reduce the voltage stress on switches and decrease the generation of harmonics.
- Implementing direct torque control techniques can help reduce the torque ripple content with the proper selection of voltage vectors and sector organization.
- Development of integrated converters effectively used in SRM drive to analyse the performance of speed, torque, and phase current at steady and dynamic conditions.

The paper is organized as follows: Sect. “Introduction” discusses the non-linearity in the inductance and flux linkage characteristics of the switched reluctance motor. It also covers the conventional converters and the proposed modular cascade converters, detailing their modes of operation along with switching tables. Section “SRM Model and modular converters” presents the proposed modular converter-fed direct torque control strategy, including the selection of voltage vectors and sector partitioning. The proposed model is developed and validated using MATLAB/Simulink, and the results of speed, phase current, and torque are analysed under various speed and dynamic conditions. Section “MCC with direct torque control (DTC)” focuses on the hardware setup and experimental results, analysing the system’s response at different operating speeds. Finally, the results are discussed, and conclusions are drawn.

## SRM model and modular converters

**Fig. 1:**
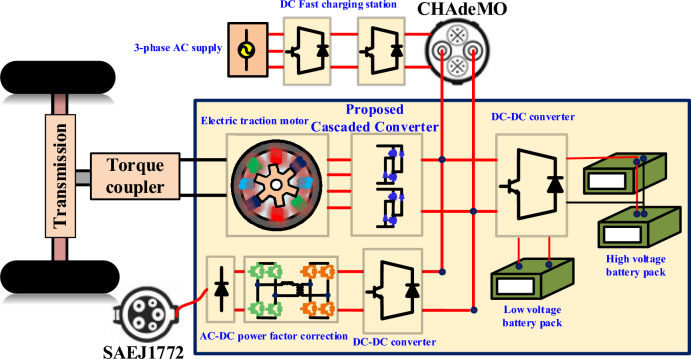
SRM motor integrated with MCC powertrain module.

The SRM converter regulates the current in each winding phase to operate the motor in different conditions, such as battery charging, AC charging, and DC fast charging, as shown in Fig. 1. The SRM model of non-linear parameters, such as flux linkage, Inductance, and rotor position, is used to identify an accurate mathematical model. Thus, the model fed from the SRM converters operates the motor with good performance. Torque production adjusts the phase currents to produce the required torque, optimizing performance across different speed ranges. Switching coordination precisely controls the switching of phase windings to minimize torque ripple and maximize efficiency. The SRM converters play a crucial role in operating motors in different operations in EVs, such as speed and torque control and charging. The accurate mathematical model of SRM parameters is important to design a controller to get proper switching of the modular converters.

The flux linkage can be calculated from the single-phase voltage equation expressed as

1 $$v = R_{s} i_{j} + \frac{{\partial \psi_{s} (\theta ,i)}}{\partial t}.$$

When the phase current equation is remodified from (1), it can be expressed as

2 $$\frac{di}{{dt}} = \left( {\frac{\partial \psi (\theta ,i)}{{\partial i}}} \right)^{ - 1} .\left( {v - R_{s} i_{j} - \frac{\partial \psi (\theta ,i)}{{\partial \theta }}\frac{d\theta }{{dt}}} \right).$$

The torque can be expressed as

3 $$T_{e} = \sum\limits_{j = 1}^{4} {T_{j} } (i_{j} ,\theta_{j} ).$$

**Fig. 2:**
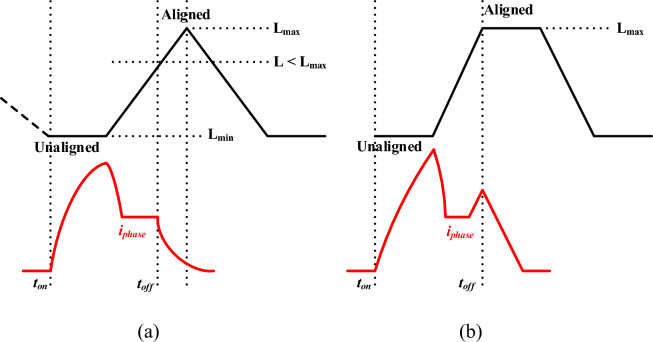
SRM pole alignment (**a**) Rotor pole equals stator pole, (**b**) Rotor pole greater than stator pole.

**Fig. 3:**
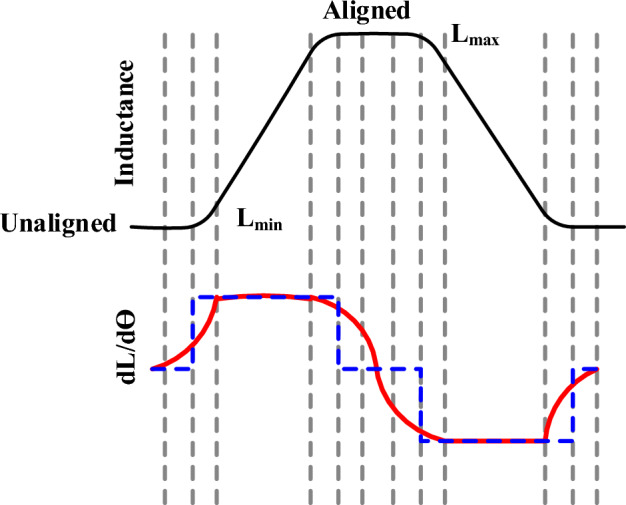
Inductance profile SRM with two positions.

The double salient pole SRM generates torque production with the alignment of the stator and rotor poles. Where the stator pole is equal to the rotor pole, torque production creates high oscillation, and a high rotor pole creates smooth torque production, as shown in Fig. 2. Thus, the unaligned Inductance represents the lowest Inductance and highest reluctance, producing useful torque. Aligned Inductance is the maximum inductance state with no torque production since the rotor is fully aligned as shown in Fig. 3. Maintaining an optimal current level in each phase during its excitation period is essential for maximizing torque.

**Fig. 4:**
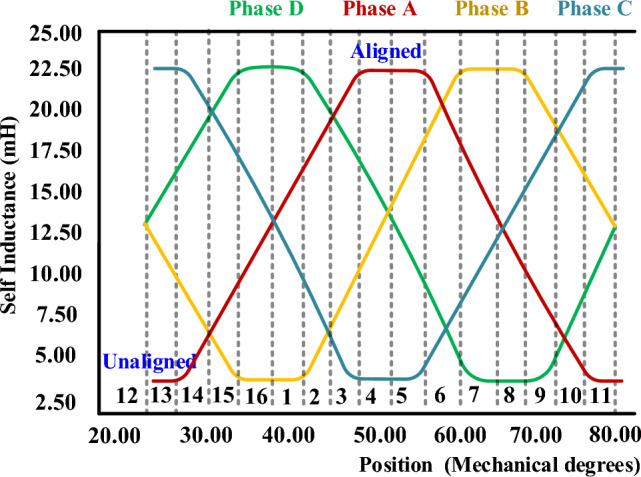
SRM four-phase rotor position and Inductance.

The SRM position relative to the stator windings significantly affects the Inductance and torque generation for all four phases, as shown in Fig. 4. Torque in an SRM is generated due to the rotor’s natural tendency to move towards a position of higher Inductance (lower reluctance) when a phase winding is energized. The 8/6 SRM input supply of all four phases with rotor position, aligned Inductance, unaligned Inductance, and torque production for each interval is listed in Table 1. The phase excitation and switching pattern for 180° electrical rotation for half-cycle symmetry. The total Inductance is calculated as L_1_L_2_/L_1_ + L_2_.

**Table 1:** Four-phase SRM conduction with inductance.

Rotor position (electrical degrees)	Phase A	Phase B	Phase C	Phase D	Unaligned inductance	Aligned inductance	Maximum torque production
0°	P	N	N	N	A	C	+ T_A_−T_C_
45°	N	P	N	N	B	D	+ T_B+_ T_D_
90°	N	N	P	N	C	A	+ T_C+_T_A_
135°	N	N	N	P	D	B	+ T_D_−T_B_
180°	P	N	N	N	A	C	−T_C+_T_A_
225°	N	P	N	N	B	D	−T_D+_T_B_
270°	N	N	P	N	C	A	−T_A+_T_C_
315°	N	N	N	P	D	B	−T_B_- + T_D_

Energization ‘P’, Demagnetization ‘N’.

The above table indicates the switching pattern and torque production of the phases during each interval. The actual electromagnetic torque depends on the inductance profile and rotor position.

**Fig. 5:**
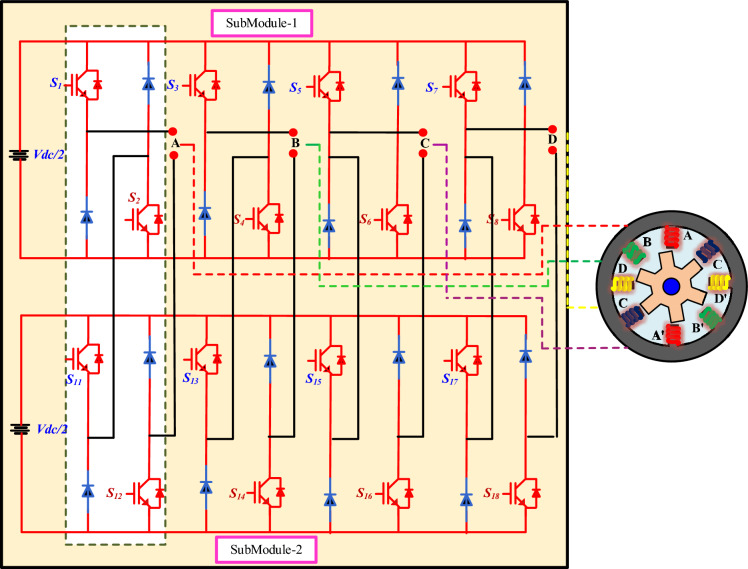
Functional block diagram of MCC-fed SRM.

The Modular multiport Cascaded Converter (MCC) topology is used to control a four-phase SRM, as shown in Fig. 5. This converter architecture consists of MCC cells connected in series per phase.

Two cascaded H-bridge submodules drive each phase (A, B, C, and D). Each H-bridge consists of four IGBTs, with anti-parallel diodes, and is either powered by an independent DC source. One cascaded MCC submodule produces three voltage levels: + Vdc, 0, and −Vdc. The three identical submodules connected in series; the output voltage is the sum of the individual submodule voltages. Therefore, In the proposed cascading structure of two submodules, the system can produce five distinct output voltage levels: ± Vdc, ± Vdc/2, and 0.

This multilevel voltage generation significantly improves the quality of the output waveform, reduces total harmonic distortion (THD), and minimizes stress on the motor insulation due to lower dv/dt. The modular nature of the converter allows for scalability and fault tolerance, as each bridge operates independently and can be bypassed if a fault occurs. The connection from the converter to the motor phases ensures controlled excitation of each stator winding, enabling precise control of torque and speed. Advanced control strategies such as phase-shifted PWM, selective harmonic elimination, or model predictive control can be used to modulate the switches, improving performance in terms of efficiency, torque ripple reduction, and dynamic response.

**Fig. 6:**
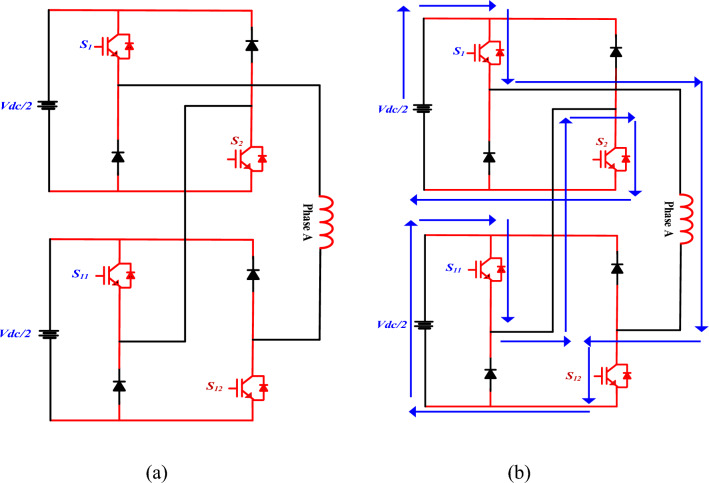
Single phase MCC (**a**) phase A connection, (**b**) Mode 1.

The switches $S_{1}$ and $S_{2}$ of the main converter, and $S_{11}$ and $S_{12}$ of the auxiliary converter are turned ON as shown in Fig. 6, resulting in a + Vdc voltage level at the converter output terminals (Table 2).

**Table 2:** MCC switching operation.

Mode of operations	Switching	Output voltage	Operation mode
Mode 1	S1,S12,S11,S2 ON	+ Vdc	Magnetization
Mode 2	S1,S2,S12 ON	+ Vdc/2	Magnetization
Mode 3	S2,S11,S1 ON	− Vdc/2	Demagnetization
Mode 4	S2,S11 ON	0	Free wheeling
Mode 5	S2,S11,S12,S1 ON	− Vdc	Demagnetization

**Fig. 7:**
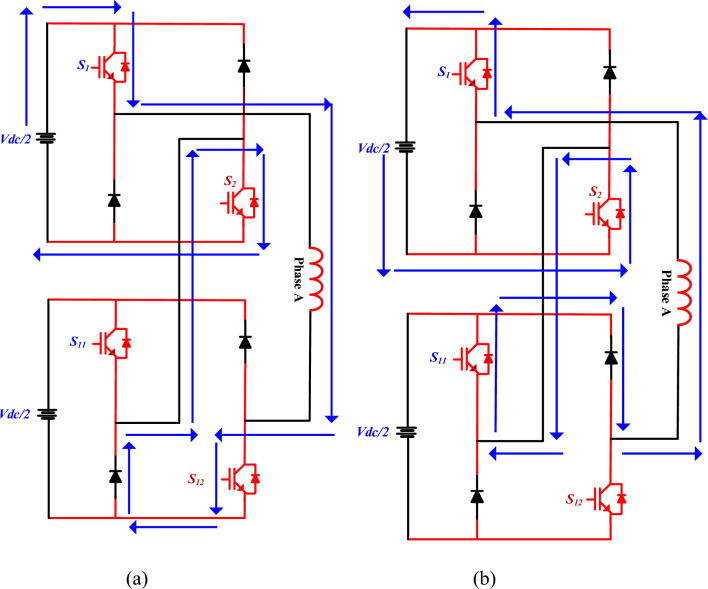
Single phase MCC (a) Mode 2, (b) Mode 3.

As illustrated in Fig. 7, specific switches of the main converter and auxiliary converter are turned ON to generate a positive voltage level at the output terminals. The complementary switches are turned OFF, which in turn activates the freewheeling diodes present in their respective legs. This diode conduction clamps the corresponding main converter switch, effectively establishing a + V_dc_/2 voltage level at the converter output. This clamping mechanism plays a key role in regulating the output voltage while ensuring proper energy flow through the converter.

**Fig. 8:**
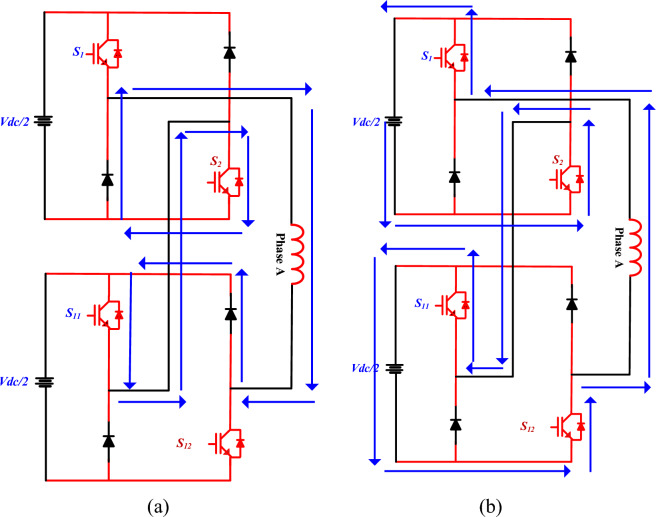
Single phase MCC (a) Mode 4, (b) Mode 5.

Figure 8a shows the case where the auxiliary converter switches (S_2_) and the main converter switch (S_11_) are turned ON. However, the complementary action causes the associated diodes in the auxiliary legs to conduct. This condition helps in generating either a neutral or clamped voltage level at the converter output, depending on the specific modulation scheme and load condition. In this mode, the proposed converter acts as a freewheeling mode. In this condition, simultaneously across different legs, resulting in a net output of zero voltage at the converter terminals. This zero-voltage output condition is crucial for implementing multilevel voltage balancing and minimizing switching losses during specific modulation intervals.

In Fig. 8b, another switching state is demonstrated, where selected switches of both the main and auxiliary converters are turned ON. The complementary diodes are turned OFF, forcing the corresponding switches to conduct. This coordinated switching conduction enables the converter to synthesise both −V_dc_/2.

As shown in Fig. 8, when the switches of both the main and auxiliary converters are turned OFF, the complementary diodes in their respective legs are forced to conduct. This diode conduction provides a specific voltage level at the output terminals of the converter, enabling controlled voltage shaping in accordance with the desired output waveform.

A basic closed-loop configuration typically includes a PI controller for speed regulation, integrated with a current control loop to manage phase excitations. In line with this approach, a closed-loop control strategy was implemented for the proposed cascaded converter to regulate motor speed and improve system performance.

At the heart of the system lies the MCC, which converts the regulated DC voltage into controlled AC waveforms suitable for energising the SRM phases. A crucial component of control strategy is the accurate regulation of phase currents, which directly influences torque generation and dynamic response. Therefore, the next section discusses the advanced control techniques with good dynamic response of SRM.

## MCC with direct torque control (DTC)

DTC is an advanced technique used to control the torque and flux of a motor directly by selecting appropriate voltage vectors. In the context of an MMCC used in switched SRM drives, the application of DTC involves the use of eight voltage vectors and eight sectors to control the switching of the converter in a way that optimises performance.

DTC controls the torque and flux by selecting optimal voltage vectors based on the current state of the motor. The key components of DTC are the torque and flux estimators, a sector determination block, and a switching table that selects the voltage vectors. The eight voltage vectors represent different states the converter can switch to adjust the motor’s electromagnetic torque and stator flux linkage. The stator flux linkage space is divided into eight sectors (typically numbered from 1 to 8). The MCC generates eight voltage vectors (VVs), each corresponding to a different switching state of the converter. These VVs are labelled (*V*_*0*_*, V*_*1*_*, V*_*2*_*, …, V*_*7*_) and are selected based on the motor’s torque and flux requirements. The chosen vector is then applied to the MMCC, adjusting the converter’s output voltage to drive the SRM phase windings. The switching table is crucial as it maps the torque and flux error signals with the current sector to select the optimal VVs.

4 $$\psi_{1} = \int {(v_{a} } - r_{a} .i_{a} ).dt.$$

**Fig. 9:**
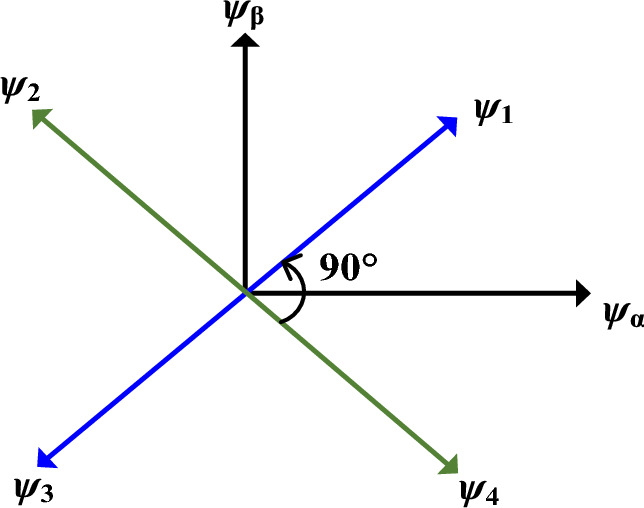
DTC orthogonal reference frame.

The 8/6 SRM, four fluxes as shown in Fig. 9, can be transformed to an orthogonal reference frame ($\psi_{\alpha } ,\psi_{\beta }$) flux are

5 $$\psi_{\alpha } = \psi_{1} \cos 45^{ \circ } - \psi_{2} \cos 45^{ \circ } - \psi_{3} \cos 45^{ \circ } + \psi_{4} \cos 45^{ \circ } ,$$

6 $$\psi_{\beta } = \psi_{1} \sin 45^{ \circ } + \psi_{2} \sin 45^{ \circ } - \psi_{3} \sin 45^{ \circ } - \psi_{4} \sin 45^{ \circ } .$$

The magnitude and angle of the flux is

7 $$\left| {\psi_{s} } \right| = \left( {\psi_{\alpha }^{2} + \psi_{\beta }^{2} } \right)^{\frac{1}{2}} ,$$

8 $$\angle \psi_{s} = \arctan \left( {\frac{{\psi_{\beta } }}{{\psi_{\alpha } }}} \right).$$

**Fig. 10:**
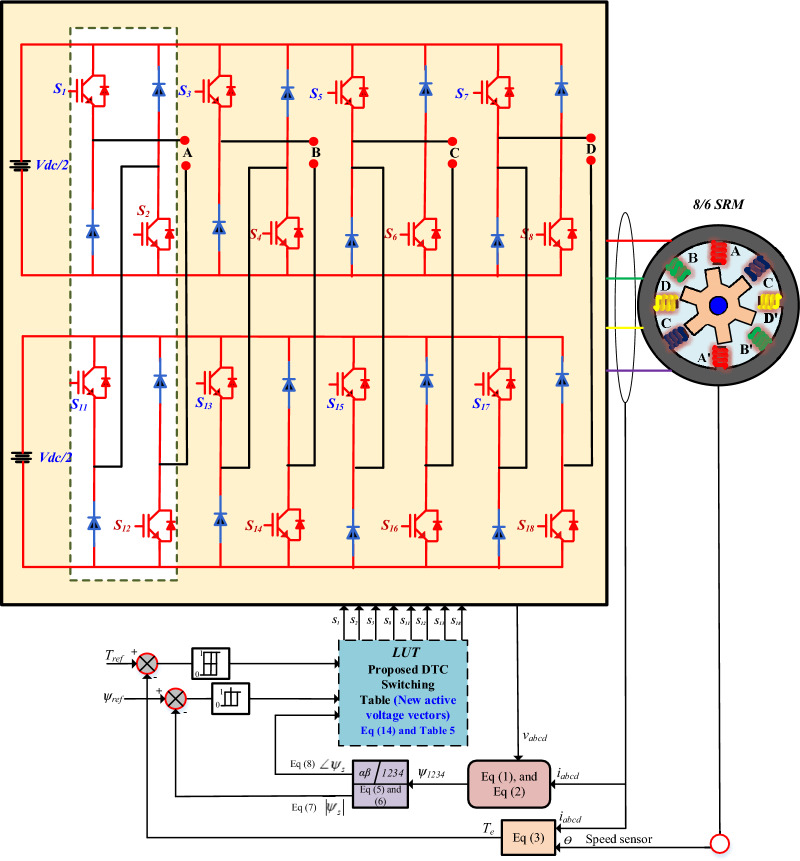
Proposed functional block diagram MCC-SRM driven DTC.

This table typically has entries that specify which voltage vector to apply based on whether the torque and flux errors are positive, negative, or zero. The integration of DTC techniques with an MCC in an SRM drive leverages eight voltage vectors and eight sectors to dynamically control the motor as shown in Fig. 10.

**Fig. 11:**
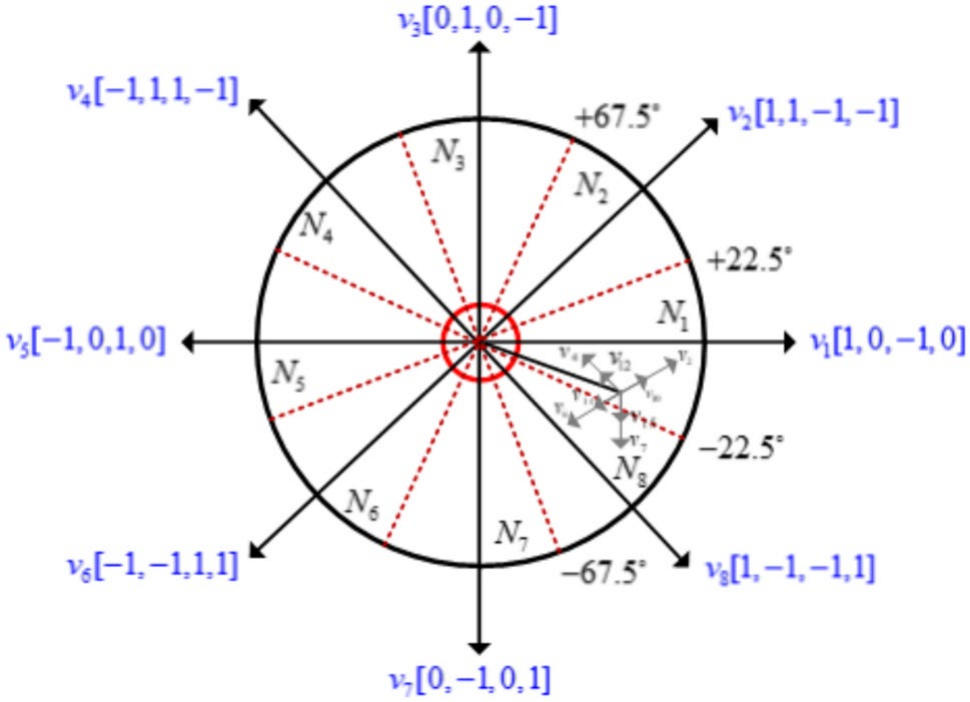
Proposed DTC VVs and sector organization.

The DTC techniques selection of VVs and sectors is partitioned as shown in Fig. 11. In this case, the selection of candidate VVs is eight from (V_1_ to V_8_) at high VVs magnitude with 45°. The sectors are selected from eight (N_1_ to N_8_). The eight VVs and sectors are the best selection to estimate the torque and flux within the band to choose the optimal switching states.

The continuous selection of voltage vectors based on real-time torque and flux errors ensures optimal switching states as listed in Table 3.

**Table 3:** Switching strategy.

Sectors	Torque	Flux	Selected VVs
*N*_*1*_	+ + − −	+ − + −	V_1_,V_2_,V_3_,V_4_
*N*_*2*_	+ + − −	+ − + −	V_2_,V_3_,V_4_,V_5_
*N*_*3*_	+ + − −	+ − + −	V_3_,V_4_,V_5_,V_6_
*N*_*4*_	+ + − −	+ − + −	V_4_,V_5_,V_6_,V_7_
*N*_*5*_	+ + − −	+ − + −	V_5_,V_6_,V_7_,V_8_
*N*_*6*_	+ + − −	+ − + −	V_6_,V_7_,V_8_,V_1_
*N*_*7*_	+ + − −	+ − + −	V_7_,V_8_,V_1_,V_2_
*N*_*8*_	+ + − −	+ − + −	V_8_,V_1_, V_2_, V_3_

The switching table in DTC is a crucial decision-making tool that directly controls the voltage vector applied to the SRM drive. By continuously selecting the optimal vector based on real-time torque, flux, and sector information, the system achieves smooth, responsive, and efficient control over the motor, enhancing performance while minimizing losses.

## Result and discussion

The simulation of the SRM drive was performed using MCC-DTC techniques in MATLAB/Simulink to validate the drive’s performance. The proposed model parameters are derived and listed in Table 4. This parameter helps to model the motor integrated with MCC-DTC in the MATLAB model-based design. The simulation scenarios closely replicated the experimental conditions to provide a comparative analysis between simulated and real-world behaviour. The key aspects analyzed were speed, torque, and phase current responses, with a focus on evaluating the torque ripple during different operating states.

**Table 4:** Parameters of SRM drives.

Motor	Parameters
Resistance	2.9 Ω
Inertia	0.079 kg. m.m
Friction	0.023 N.m. s
Flux linkage	0.684 Wb
Maximum inductance	3.58 mH
Minimum inductance	0.57 mH
Saturated inductance	2.27 mH

**Fig. 12:**
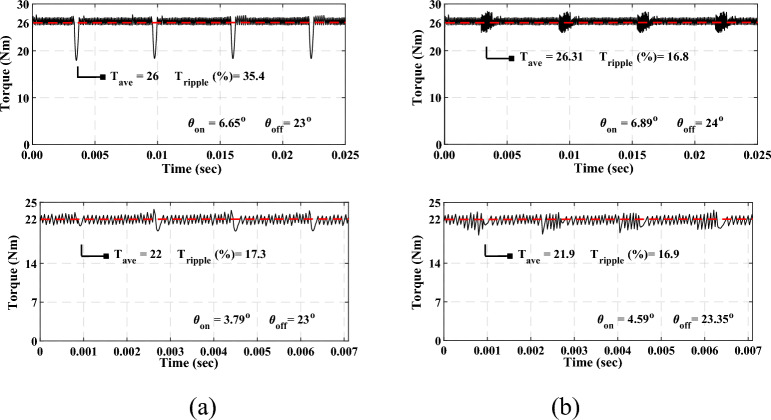
Simulation results (**a**) Conventional MCC-based SRM, (**b**) Proposed MCC-DTC.

In the simulation results shown in Fig. 12, the SRM drive operates at a steady-state speed of 1000 rpm for both the conventional MCC and the proposed MCC-DTC. The speed response remains stable with negligible oscillations, confirming the effectiveness of the DTC method in maintaining the target speed. In Fig. 12a, the speed is maintained at 1000 rpm, and the average torque is 26 Nm. Under a torque load condition of 26 Nm, the drive delivers 16.8 Nm, indicating partial torque delivery. The torque response shows noticeable fluctuations, reflecting the dynamic control of the electromagnetic torque. The phase current waveform remains consistent, with balanced excitation and low ripple. The simulated torque ripple is calculated to be approximately 35.4% and 17.3% for different modes. Figure 12a also shows balanced phase current waveforms with moderate ripple, indicating effective current regulation and smooth commutation. The torque-to-current ratio (T_ave_/I_rms_ = 1.67) and system efficiency of 84.37% confirm optimal current utilization and efficient drive performance. The SRM drive was subjected to the same conditions for the proposed techniques for MCC-DTC at 1000 rpm to analyse its acceleration performance. During acceleration, the speed increased and decreased smoothly with a short transient period, and the drive quickly settled at the target speeds of 1000 rpm and 500 rpm. In Fig. 12b, the speed is maintained at 1000 rpm, and the average torque is 22 Nm. Under a torque load condition of 26 Nm, the drive delivers 16.9 Nm, indicating partial torque delivery. The torque response shows noticeable fluctuations, reflecting the dynamic control of the electromagnetic torque. The phase current waveform remains consistent, with balanced excitation and low ripple. The simulated torque ripple is calculated to be approximately 35.4% and 16.9% for different modes. Figure 12a also shows balanced phase current waveforms with moderate ripple, indicating effective current regulation and smooth commutation. The torque-to-current ratio (T_ave_/I_rms_ = 1.46) and system efficiency of 84.55% confirm optimal current utilization and efficient drive performance.

**Fig. 13:**
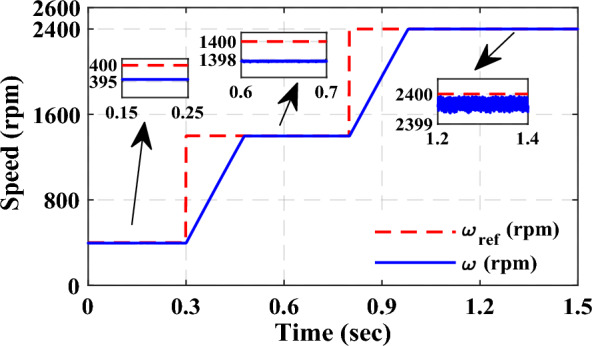
Proposed MCC-based DTC-SRM Dynamic speed changes.

Overall, the proposed MCC-DTC offers better dynamic response, lower torque ripple, and slightly improved efficiency, making it more suitable for high-performance SRM drive applications despite a marginally lower torque-to-current ratio. The dynamic speed response of the SRM drive using the proposed MCC-DTC strategy is illustrated in Fig. 13. The drive is subjected to multiple speed commands, 400 rpm, 1400 rpm, and 2400 rpm, to evaluate its performance under varying operating conditions.

Initially, the drive settles at 395 rpm for a reference of 400 rpm, showing minimal steady-state error. When the speed is stepped up to 1400 rpm, the motor quickly accelerates and stabilizes at 1398 rpm with a short transient period and no overshoot. However, the speed increase to 2400 rpm is also accurately tracked, with the actual speed settling around 2390 rpm.

**Fig. 14:**
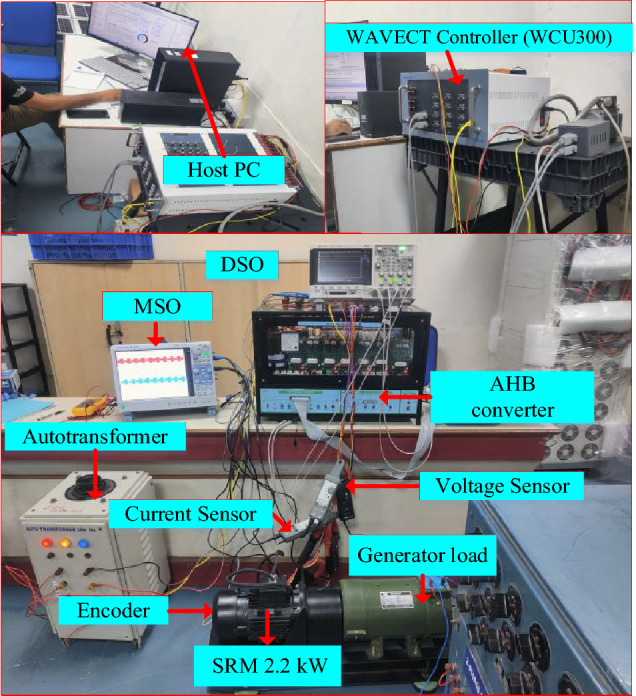
Experimental hardware setup MCC with 8/6 SRM drive.

The experimental setup of the proposed MMCC-fed SRM with DTC, as shown in Fig. 14, is built to analyze various performance parameters. The MCC incorporates Semikron IGBT switches, voltage and current sensors, and an encoder to measure rotor position. The DTC is developed using a model-based design and is interfaced with an 8/6 SRM rated at 2.2 kW.

**Fig. 15:**
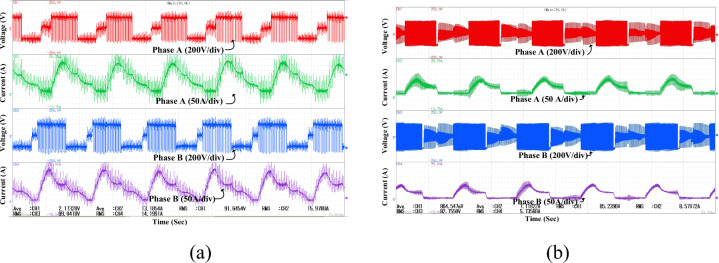
Experimental results (**a**) Conventional MCC (**b**) Proposed MCC-DTC.

Figure 15a presents the experimental waveforms of phase A and phase B voltages and currents under steady-state operation of the SRM drive using the conventional MCC. The phase currents exhibit significant distortion and high ripple, indicating limited current regulation and commutation effectiveness. Although the waveforms show some symmetry, the presence of high ripple reflects unbalanced phase excitation. In contrast, Fig. 15b shows the waveforms under identical operating conditions using the proposed MCC-DTC controller. Here, the current waveforms demonstrate good symmetry and low ripple, indicating balanced phase excitation and precise current control. These results validate the practical implementation of the proposed control strategy and confirm that the inverter delivers appropriate voltage pulses for achieving stable and efficient SRM operation.

**Fig. 16:**
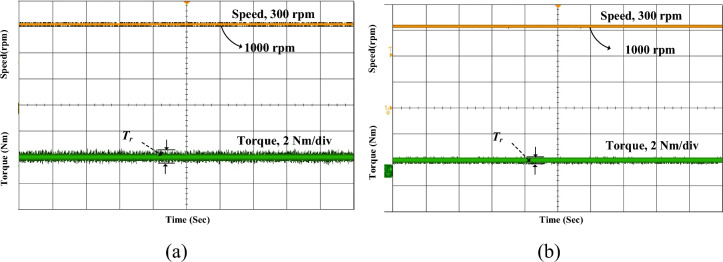
Experimental for steady state (**a**) Conventional (**b**) Proposed MCC-DTC.

Figure 16 presents the experimental results of the SRM drive under steady-state conditions at a constant speed of 1000 rpm for both conventional MCC and the proposed MCC-DTC. In both cases, the reference speed is accurately maintained, indicating good speed regulation. However, a clear difference is observed in the torque response. Additionally, the torque waveform in the proposed method shows reduced ripples compared to the conventional approach, indicating smoother and more stable steady-state operation.

**Fig. 17:**
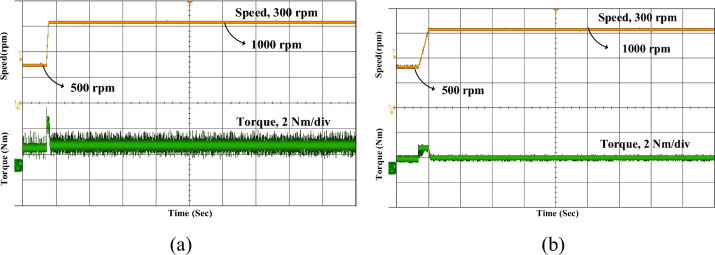
Experimental speed acceleration (**a**) Conventional (**b**) Proposed MCC-DTC.

The experimental acceleration speed is commanded to rise from 500 to 1000 rpm, as shown in Fig. 17. With the conventional MCC, the actual speed gradually increases with a relatively longer settling time and exhibits higher oscillations and ripple during the transient period. In contrast, the proposed MCC-DTC controller demonstrates a faster and smoother acceleration profile, while the torque response remains stable with significantly lower ripple.

**Fig. 18:**
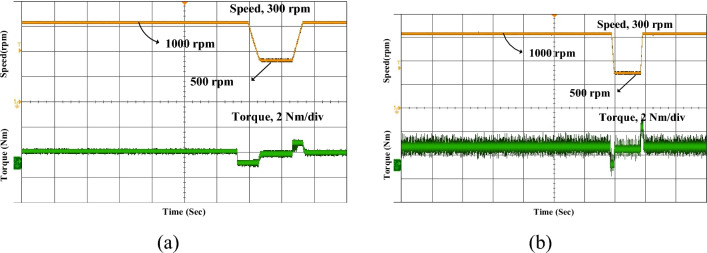
Experimental speed deceleration (**a**) Conventional (**b**) Proposed MCC-DTC.

Figure 18 illustrates the experimental deceleration response of the SRM drive when the speed is reduced from a higher reference to 500 rpm. In conventional, the torque waveform exhibits noticeable ripple and instability, which may result in mechanical stress and uneven braking performance. Therefore, the proposed MCC-DTC demonstrates a faster and smoother deceleration, with significantly lower ripple compared to the conventional method. Overall, the results confirm the robustness and responsiveness of the MCC-DTC strategy in both acceleration and deceleration phases of motor operation.

**Fig. 19:**
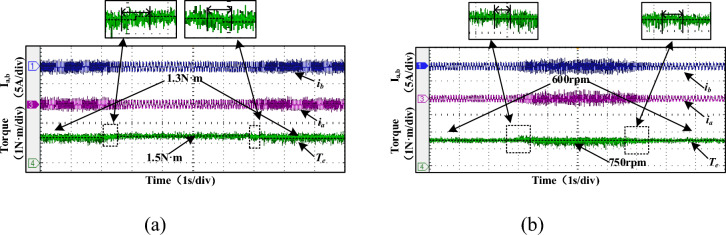
Experimental proposed MCC-DTC (**a**) Loading and unloading (**b**) Transient conditions.

The experimental validation of the proposed MCC-DTC controller under loading and unloading, and transient operating conditions are shown in Fig. 19. The current and speed waveforms remain well-regulated, demonstrating the robustness of the proposed control strategy in real-time load disturbances. Under transient conditions, such as speed or torque reference changes, the MCC-DTC ensures rapid response with minimal overshoot and fast settling. The drive quickly adapts to new setpoints while maintaining system stability, indicating excellent dynamic performance. These results confirm that the proposed MCC-DTC not only enhances steady-state behavior but also performs reliably under dynamic and unpredictable operating environments, making it well-suited for practical SRM drive applications. Table 5 compares the performance of the conventional MCC and the proposed MCC-DTC in terms of ripple at different speeds and under load conditions. At a low speed of 500 rpm, the conventional MCC exhibits a ripple of 35.4%, whereas the proposed MCC-DTC reduces it significantly to 25.4%, demonstrating better low-speed torque control.

**Table 5:** Performance on different speed responses.

Performance	Conventional MCC	Proposed MCC-DTC
	Torque ripple, %	
500 rpm	35.4	25.4
1500 rpm	26.5	19.6
Torque load, Nm	23.7	16.3

Similarly, at 1500 rpm, the ripple is further reduced from 26.5% with conventional MCC to 19.6% using MCC-DTC, indicating improved performance across a wider speed range. Under a torque load condition, the proposed MCC-DTC achieves a much lower ripple of 16.3% compared to 23.7% in the conventional approach.

**Fig. 20:**
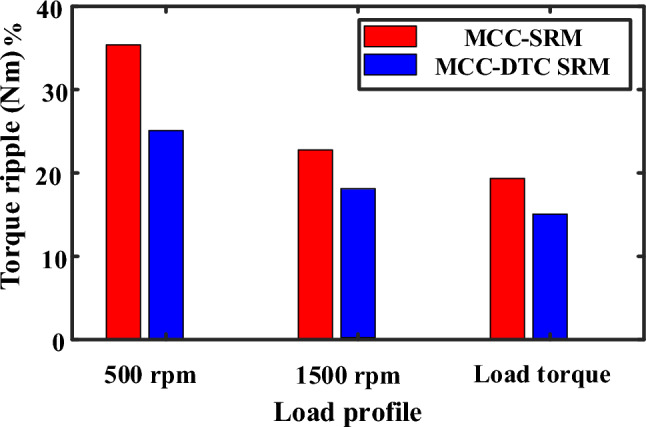
Torque ripple performance.

Both simulation and experimental results exhibit excellent speed control with minimal deviation during steady-state and dynamic conditions as shown in Fig. 20. The torque ripple values from simulations closely match experimental results, validating the proposed MCC-DTC effectiveness in both environments. These results clearly highlight the effectiveness of the proposed MCC-DTC method in minimizing torque ripple, enhancing drive smoothness, and ensuring better electromagnetic torque regulation, especially under dynamic and loaded conditions.

The proposed MCC-DTC method demonstrates clear performance advantages over the conventional MCC approach as listed Table 6. It achieves very fast dynamic response and significantly reduces torque ripple, ensuring smoother operation. By employing a fixed switching frequency, it enhances predictability and control stability, while maintaining simple logic that reduces algorithmic complexity. However, these benefits come at the expense of higher cost and hardware requirements compared to the conventional MCC, which, despite being more economical, suffers from slower response, higher torque ripple, and variable switching frequency that limit its suitability for demanding applications.

**Table 6:** Comparison of the proposed DTC and conventional.

Control method	Proposed MCC-DTC	Conventional MCC
Dynamic response	Very fast	Slow response
Torque ripple	Lower ripple	High ripple
Switching frequency	Fixed switching frequency	Variable switching frequency
Complexity	Simple logic	High
Cost and hardware	High	Low

## Conclusion

The simulation and experimental validation of the proposed MCC-DTC control strategy for SRM drives has demonstrated significant improvements in both steady-state and dynamic performance compared to the conventional MCC approach. The proposed method effectively reduces torque ripple, enhances current waveform quality, and ensures precise speed regulation across various operating conditions, including loading, unloading, acceleration, and deceleration.

- The torque ripple values were significantly reduced to 25.4%, 19.6%, and 16.3% under various operation conditions.
- Notably, the MCC-DTC controller offers faster transient response with minimal overshoot and settling time, along with improved torque control and system stability.

These improvements confirm that MCC-DTC is a reliable and high-efficiency control strategy suitable for real-time SRM drive applications.

## Data Availability

The data that support the findings of this study are available from Centre for Electric Mobility (CEM) SRM Institute of Science and Technology, India but restrictions apply to the availability of these data, which were used under license for the current study, and so are not publicly available. Data are however available from the authors upon reasonable request and with permission of Centre for Electric Mobility ( CEM) , SRM Institute of Science and Technology, India Specifically, the data related to SRM motor controller development and the implementation of direct torque control (DTC) techniques—used to minimize torque ripple through optimized voltage vector and sector selection—are maintained at CEM, SRM Institute of Science and Technology, India. As these data form part of an ongoing industry project, they cannot be shared publicly. However, results-based portions of the dataset may be shared upon reasonable request, subject to institutional approval and with the objective clearly stated.

## References

[1] Mohanraj, D., Gopalakrishnan, J., Chokkalingam, B. & Mihet-Popa, L. Critical aspects of electric motor drive controllers and mitigation of torque ripple—review. *IEEE Access* **10**, 73635–73674. 10.1109/ACCESS.2022.3187515 (2022).

[2] Gobbi, M., Sattar, A., Palazzetti, R. & Mastinu, G. Traction motors for electric vehicles: Maximization of mechanical efficiency–a review. *Appl. Energy* **357**, 122496 (2024).

[3] Kosuru, V. S. R. & Venkitaraman, A. K. Trends and challenges in electric vehicle motor drivelines-a review. *Int. J. Electr. Comput. Eng. Syst.* **14**(4), 485–495 (2023).

[4] Rimpas, D. et al. Comparative review of motor technologies for electric vehicles powered by a hybrid energy storage system based on multi-criteria analysis. *Energies* **16**(6), 2555 (2023).

[5] Song, Z. & Liang, Y. Overview of high overload motors. *IEEE Trans. Ind. Appl.* **60**(6), 8611–8626 (2024).

[6] Teymoori, V., Kamper, M., Wang, R.-J. & Kennel, R. Sensorless control of dual three-phase permanent magnet synchronous machines—a review. *Energies* **16**(3), 1326 (2023).

[7] Thirumalasetty, M. & Narayanan, G. High-performance torque controller for switched reluctance machine. *IEEE Trans. Ind. Appl.* **60**, 6923–6937 (2024).

[8] Gaafar, M. A., Abdelmaksoud, A., Orabi, M., Chen, H. & Dardeer, M. Switched reluctance motor converters for electric vehicles applications: Comparative review. *IEEE Trans. Transp. Electrif.* **9**(3), 3526–3544 (2022).

[9] Khalid, H. et al. A high voltage gain multi-stage DC-DC boost converter with reduced voltage stress. *IETE J. Res.* **70**(2), 2032–2046 (2024).

[10] Kumar, A., Singh, B. & Singh, G. Modified direct torque control of solar fed sensorless switched reluctance motor drive for electric vehicle with regenerative braking. *IEEE Trans. Ind. Appl.* **60**, 3155–3154 (2023).

[11] Shakeri, S., Koochi, M. H. R. & Esmaeili, S. Optimal harmonic resonance monitoring in electrical network considering area of harmonic pollution and system uncertainty. *IET Gener. Transm. Distrib.* **18**, 2570–2586 (2024).

[12] Mohanraj, D., Umavathi, M., Verma, R., Chokkalingam, B. & Mihet-Popa, L. Enhancing performance toward torque and flux control through a hybrid approach of intelligent and DTC for SRM drives. *IEEE Access* **13**, 92168–92179 (2025).

[13] Deepak, M., Janaki, G., Bharatiraja, C. & Ojo, J. O. An enhanced model predictive direct torque control of SRM drive based on a novel modified switching strategy for low torque ripple. *IEEE J. Emerg. Sel. Top. Power Electron.* **12**, 2203–2213 (2023).

[14] Samithas, D. et al. Experimental analysis of enhanced finite set model predictive control and direct torque control in SRM drives for torque ripple reduction. *Sci. Rep.* **14**, 1–19 (2024). 39039123 10.1038/s41598-024-65202-1 PMC11263375

[15] Das, D. et al. Optimal design of a novel modified electric eel foraging optimization (MEEFO) based super twisting sliding mode controller for controlling the speed of a switched reluctance motor. *Sci. Rep.* **14**, 32006 (2024). 39738758 10.1038/s41598-024-83495-0 PMC11685943

[16] Deepak, M., Bharatiraja, C., Williamson, S. S. & Krishnamurthy, M. Enhanced direct torque control of SRM based on a novel multilevel hysteresis torque band with effective voltage vectors for low torque ripple. *IEEE Trans. Transp. Electrif.* **1**, 12758–12770 (2025).

[17] Phukan, R. et al. Characterization and mitigation of conducted emissions in a SiC based three-level T-type motor drive for aircraft propulsion. *IEEE Trans. Ind. Appl.* **59**(3), 3400–3412 (2023).

[18] Wireko-Brobby, A. et al. Analysis of the sources of error within PMSM-based electric powertrains-a review. *IEEE Trans. Transp. Electrif.* **10**, 6370–6406 (2023).

[19] Schenke, M., Haucke-Korber, B. & Wallscheid, O. Finite-set direct torque control via edge computing-assisted safe reinforcement learning for a permanent magnet synchronous motor. *IEEE Trans. Power Electron.* **38**, 13741–13756 (2023).

