# Correction: Performance-driven switched reluctance motor drive using multiport cascaded converter and advanced direct torque control scheme

**DOI:** 10.1038/s41598-026-57370-z

**Published:** 2026-06-18

**Authors:** M. Deepak, C. Santhakumar, K. Sathiyasekar, Bharatiraja Chokkalingam, Sanjeevikumar Padmanaban

**Affiliations:** 1 Department of Electrical and Electronics Engineering, KIT-Kalaignar Karunanidhi Institute of Technology, Coimbatore, India; 2 Department of Electrical and Electronics Engineering, K. S. R. College of Engineering, Tiruchengode, India; 3 Centre for Electric Mobility, Department of Electrical and Electronics Engineering, SRM Institute of Science and Technology, Kattankulathur, Chengalpattu, Tamil Nadu 603203 India; 4 Department of Electrical Engineering, Information Technology and Cybernetics, University of South-Eastern Norway, Kongsberg, Norway

Correction to: *Scientific Reports* 10.1038/s41598-026-45141-9, published online 17 March 2026

The original version of this Article contained an error in the spelling of the author C. Santhakumar which was incorrectly given as K. Santhakumar.

The original Article has been corrected.

